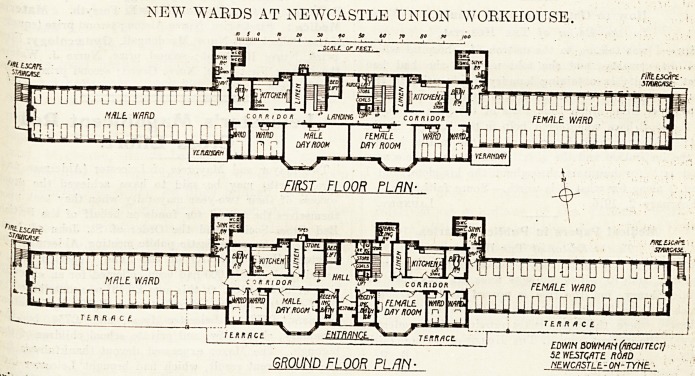# Reports on Hospitals of the United Kingdom

**Published:** 1916-02-12

**Authors:** Henry Burdett


### REPORTS ON
### Hospitals of the United Kingdom.

By SIR HENRY BURDETT, K.C.B., K.C.V.O.

### THE NEW HOSPITAL WARDS AT NEWCASTLE UNION WORKHOUSE.

THE Chairman of the Newcastle Board of Guardians was kind enough to ask the members of a Conference at Newcastle to visit the new hospital wards at this workhouse. We regretted to find that only three other members of the Conference thought it worth while to accept the invitation, and we can assure those who omitted to do so that they lost a most useful opportunity for practical instruction in many matters relating to hospital construction and the latest ideas, which they would have valued had they known of them. For instance, the wards are a straight pavilion of three storeys, consisting of six principal wards with accommodation for thirty beds each. Separating the large wards on each floor are the day rooms and offices connected with each ward's unit. The wards have considerable interest from the fact that they are some twenty-four feet wide and only eleven feet high. The height, owing to the excellent windows adopted in abundance, improves the hygienic condition of these wards. This is a material fact which has a special interest at the present time, when the demand is for a reduction in the cost of hospital construction. A saving in regard to this pavilion has enabled the Guardians to complete the whole at a cost not exceeding £120 per bed. That cost could have been profitably increased to £130, with a strict eye to economy, by substituting a better kind of tessellated concrete for the relatively inferior and most unsightly type of concrete used. The same remark applies to the wood floors, which are of pine—the worst form of wood and most costly in the end of any wood which can be used for such a purpose. Canadian willow or even teak in the end would have been more economical and infinitely satisfactory in every way. On the upper floor the wards, open and in cubicles, are devoted entirely to the cases of the treatment of tuberculosis. The open wards are excellent, and the Guardians have had the wisdom not to plaster the walls, but to leave the brick exposed, which, if kept properly pointed and cleansed from carbon deposits, may become more and more attractive in the decorative sense as time passes. Altogether the Newcastle Board of Guardians are entitled to our warm congratulations and thanks for the excellent start they have made in their new hospital pavilion. There are six more, we understand, to follow, and they should most certainly, if the interests of the poor of Newcastle are to be properly considered, delay not a moment to accept contracts and erect with all possible speed a thoroughly up-to-date block for the treatment of surgical cases.

NEW WARDS AT NEWCASTLE UNION WORKHOUSE.

## Figures and Tables

**Figure f1:**